# Cases from My Note-Book

**Published:** 1884-10

**Authors:** Julius Schmitt

**Affiliations:** Rochester, N. Y.


### CASES FROM MY NOTE-BOOK.

JULIUS SCHMITT, M. D., ROCHESTER, N. Y.

CASE I. February 6th, 1883.—Mr. John H., forty-eight years old, very robust, weighs two hundred and fifty pounds with a height of five feet nine inches. Rheumatic swelling of left wrist and left big toe, bluish red, with pains, which he describes as if somebody sawed at his bones with a dull saw; ameliorated by heat of stove; *worse from sundown until sunrise;* no appetite. This condition has lasted for the last two weeks. Syphilinum$^{mm}$ (Swan) once a day, to be taken in the morning.

February 9th, 1883.—Slept the first night after taking one dose and took no more medicine, swelling disappearing gradually.

May 19th, 1884.—Got well in the course of two weeks and has had no repetition of rheumatism since.

CASE II. January 7th, 1884.—Martha K., nine years old, blonde, sick since last night, when she had a severe chill followed by heat. Status-praesens. Stitches in right lung from before backward and to right axilla, sometimes going to left lung. *Cannot lie on right side;* better on left side. Short hacking cough, during which mother *must hold right side of chest with hand. Cries before the cough.* During the night she had been delirious, and when mother lifted her out of bed on the commode, she cried anxiously, *"Don't let me fall."* Shortly before this attack, she had *a sore mouth.* Pulse, 144. Auscultation showed crepitant rales on right lower lung. Borax$^{cm}$ one dose dry on tongue. Placebo in water.

January 8th, 1884.—Was very sick for two hours after I gave her the dry powder; perspiration broke out all over and run in streams from the face and she complained of pains all over, even in arms and legs. Last night, however, she grew better and could lie on right side. To-day hardly any pains. Pulse, 96. Sits up in bed with a smiling face. Lungs entirely free from congestion. Placebo.

May 19th, 1884.—Recovered rapidly without any more medicine, and has not been sick since.

